# Machine learning modeling of plant phenology based on coupling satellite and gridded meteorological dataset

**DOI:** 10.1007/s00484-018-1534-2

**Published:** 2018-04-11

**Authors:** Bartosz Czernecki, Jakub Nowosad, Katarzyna Jabłońska

**Affiliations:** 1 Department of Climatology, Faculty of Geographical and Geological Sciences, Adam Mickiewicz University, Krygowskiego 10, 61 680 Poznań, Poland; 2 Space Informatics Lab, Department of Geography and GIS, University of Cincinnati, 219 Braunstein Hall, Cincinnati, OH 45221 USA; 3 Institute of Meteorology and Water Management - National Research Institute, Podleśna 61, 01 673 Warsaw, Poland

**Keywords:** Phenophase, Phenology modeling, BBCH scale, Machine learning, MODIS, E-OBS

## Abstract

Changes in the timing of plant phenological phases are important proxies in contemporary climate research. However, most of the commonly used traditional phenological observations do not give any coherent spatial information. While consistent spatial data can be obtained from airborne sensors and preprocessed gridded meteorological data, not many studies robustly benefit from these data sources. Therefore, the main aim of this study is to create and evaluate different statistical models for reconstructing, predicting, and improving quality of phenological phases monitoring with the use of satellite and meteorological products. A quality-controlled dataset of the 13 BBCH plant phenophases in Poland was collected for the period 2007–2014. For each phenophase, statistical models were built using the most commonly applied regression-based machine learning techniques, such as multiple linear regression, lasso, principal component regression, generalized boosted models, and random forest. The quality of the models was estimated using a k-fold cross-validation. The obtained results showed varying potential for coupling meteorological derived indices with remote sensing products in terms of phenological modeling; however, application of both data sources improves models’ accuracy from 0.6 to 4.6 day in terms of obtained RMSE. It is shown that a robust prediction of early phenological phases is mostly related to meteorological indices, whereas for autumn phenophases, there is a stronger information signal provided by satellite-derived vegetation metrics. Choosing a specific set of predictors and applying a robust preprocessing procedures is more important for final results than the selection of a particular statistical model. The average RMSE for the best models of all phenophases is 6.3, while the individual RMSE vary seasonally from 3.5 to 10 days. Models give reliable proxy for ground observations with RMSE below 5 days for early spring and late spring phenophases. For other phenophases, RMSE are higher and rise up to 9–10 days in the case of the earliest spring phenophases.

## Introduction

Phenology of the plants is mainly influenced by photoperiod and temperature (Swanton et al. 2000). Previous studies have shown that global warming determines the advance of phenological events (Bradley et al. 1999; Root et al. 2003; Menzel et al. 2006; Parmesan 2006; Cleland et al. 2007), and some of the plants currently approach their physiological limits (Iler et al. 2013). The consequences of changes in plant phenology due to climate change can create more feedbacks that alter *biogeochemical cycling and species interactions* (Melillo 2014), and may affect all Earth’s spheres (Elmendorf et al. 2016). Monitoring of phenological processes and plant reaction to currently observed climate change is therefore of high importance. Changes in timing of phenological phases are also important proxies in contemporary climate research, such that phenological data are commonly used in the reconstruction of long-time temperature time-series due to its longer coverage compared to instrumental observations (Schleip et al. 2008; Aono and Kazui 2008; Bradley 2013; Zheng et al. 2015).

This regularity is also confirmed in the case of Poland where the oldest discovered local records of phenological observations are dated back to the fifteenth or sixteenth century (Cybulski 1886). The modern and more reliable observations started after the World War II when the Polish Institute of Meteorology and Water Management established a nationwide phenological network. However, due to financial reasons, the network was abandoned from 1993 to 2005, while the newly established network of ground observations in 2006 was in several cases moved into new locations, thereby causing not only gap in the dataset but also inhomogeneities (Czernecki and Jabłońska 2016).

Partially, reconstruction of missing data is possible with the use of commonly applied statistical models based on meteorological features (McMaster and Wilhelm 1997). On the other hand, today’s observed rapid development of satellite-derived vegetation metrics may give a more reliable spatio-temporal pattern of particular phenophases and thus, may help in filling the gap on missing or erroneous observations. However, the phenological observations currently made by two different approaches (i.e., satellite-derived and ground-based observations) are often not treated as complementary sources of information because of differences between the point-wise and spatial approaches (Fisher and Mustard 2007). In spite of that, some previous research show varying potential for linking traditional observational phenology with satellite-derived vegetation metrics (Studer et al. 2007), although the potential for applying machine learning techniques in improving the accuracy of phenological models is still being underestimated or used only occasionally (Almeida et al. 2012).

Keeping the aforementioned in mind, the main aim of this study is to create and evaluate different statistical models for reconstructing and predicting the day of the year for the occurrence of selected phenological phases. The authors have also decided to evaluate the possibilities of using a wide range of statistical modeling techniques to create a synthetic archive dataset using only free of charge remote sensing and meteorological data as predictors. Therefore, it was also possible to (1) distinguish the amount of information provided by both sources of data and (2) define whether they are unrelated and contain possible sources of non-overlapping information, (3) and thus may (or may not) robustly contribute to aerobotanical and phenological research, especially in terms of phenological modeling where no long-term ground observations are available.

## Data and methods

### Phenological data

**Fig. 1:**
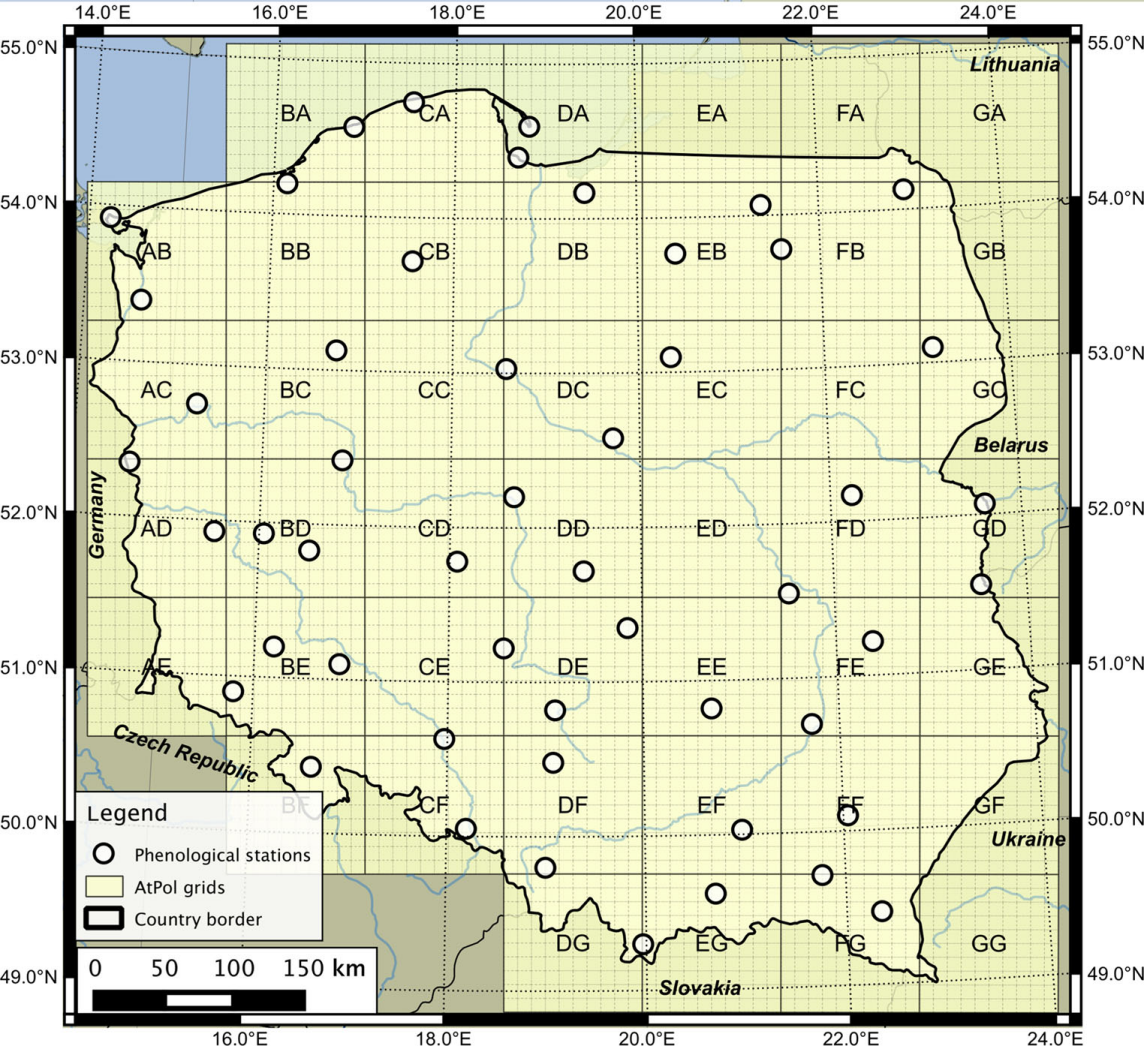
Location of selected phenological stations in Poland used in the study. Gray lines denote geobotanical AtPol (Zajac 1978; Komsta 2016) main grids (100 × 100 km; grid’s ID denoted by capital letters) and its subdivisions (10 × 10-km grids) used for aggregation of the remote sensing products

**Fig. 2:**
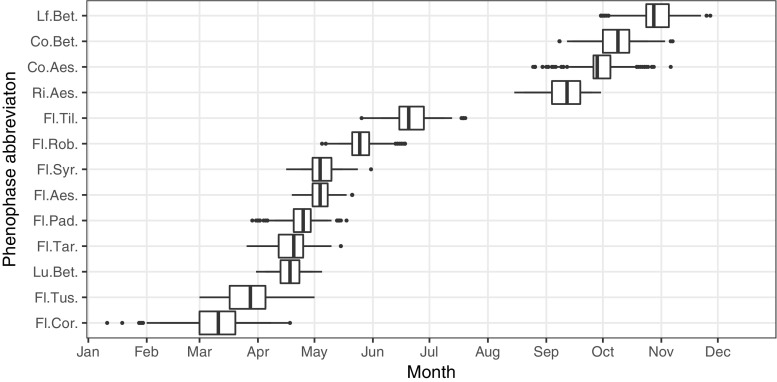
Dates of selected phenological season’s onset among all analyzed stations in Poland in the period of 2007–2014. Abbreviations for plant species as shown in Table 1. Box and whisker plots denote to percentile values of 0.05, 0.25, 0.50, 0.75, and 0.95. Outliers (i.e., values beyond percentiles 0.05–0.95) shown as dots

The study period covers the years 2007–2014 and contains dataset of ground observations of 10 species and 13 phenophases at 52 stations in Poland (Figs. 1 and 2 and Table 1). The phenological ground observation dataset used in this study originates from the newly re-established observational network run by the Institute of Meteorology and Water Management - National Research Institute (IMGW-PIB) and constitutes an important part of the national climate monitoring.

**Table 1:** Selected phenological phases, corresponding phenological seasons and abbreviations used

Species (Latin)	Species (English)	Phenophase	Phenological	No. of	Abbr.	BBCH
			season	observations		scale
*Corylus avellana* L.	Hazel	Flowering	Earliest spring	377	Fl.Cor.	BBCH 60
*Tussilago farfara* L.	Coltsfoot	Flowering	Earliest spring	377	Fl.Tus.	BBCH 60
*Betula pendula* Roth	Silver birch	Leaf unfolding	Early spring	377	Lu.Bet.	BBCH 11
*Taraxacum officinale* F.H. Wigg.	Dandelion	Flowering	Early spring	377	Fl.Tar.	BBCH 60
*Prunus padus* L.	Hackberry	Flowering	Early spring	377	Fl.Pad.	BBCH 60
*Aesculus hippocastanum* L.	Horse chestnut	Flowering	Late spring	377	Fl.Aes.	BBCH 60
*Syringa vulgaris* L.	Lilac	Flowering	Late spring	377	Fl.Syr.	BBCH 60
*Robinia pseudoacacia* L.	Black locust	Flowering	Early summer	377	Fl.Rob.	BBCH 60
*Tilia cordata* Mill.	Small-leaved lime	Flowering	Summer	347	Fl.Til.	BBCH 60
*Aesculus hippocastanum* L.	Horse chestnut	Fruit ripening	Mid autumn	298	Ri.Aes.	BBCH 86
*Aesculus hippocastanum* L.	Horse chestnut	Leaf coloring	Late autumn	287	Co.Aes.	BBCH 94
*Betula pendula* Roth	Silver birch	Leaf coloring	Late autumn	288	Co.Bet.	BBCH 94
*Betula pendula* Roth	Silver birch	Leaf falling	Late autumn	288	Lf.Bet.	BBCH 97

The phenological observational network follows the BBCH methodology (abbr. from German: “Biologische Bundesanstalt, Bundessortenamt und CHemische Industrie”), which was akin to most European countries with similar growth stages of plant species (Meier 1997; Koch et al. 2009).

To account for different levels of reliability in the data records at individual stations (due to the subjective nature of this kind of observations), and the fact that they were collected at different locations, the GIS-based kriging with external drift (Hudson and Wackernagel 1994), together with expert knowledge, was applied to detect observational outliers. Additionally, the database was revised according to several proposals regarding phenological data quality issues made by Schaber and Badeck (2002).

### Predictor variables

Three types of data sources, commonly applied in phenological modeling, were tested as potential predictors in this study:

- Preprocessed gridded meteorological data
- Satellite-derived products
- Spatial (geographical) features of monitoring sites

The rationale behind this grouping was to determine the skillful scale for each of these groups of predictors for the near-surface plant phenological modeling. The selected phenological phases might not be equally reflected in every dataset due to a different physiological reaction of plant species in selected phenophases, and hence, the different sensitivity given by remote sensing, meteorological, and spatial data. Moreover, a wide range of possible data sources with varying spatio-temporal resolution led the authors to use only free-of-charge and easy-to-access data in order to make this modeling approach applicable in all areas with similar phenological stages (e.g., in other Central European countries). Further details on feature preselection and calculated indices are described below in “Meteorological derived indices” and “Spatial features.” Brief summary of the applied predictors is included in Table 2.

**Table 2:** Summary of predictor variables used for machine learning modeling

No.	Product and its description	Predictor’s	Data	Data
		type	source	resolution
1	Altitude as derived from digital elevation model (DEM)	Spatial	SRTM-3	ca. 1 km
2–3	Geographical coordinates (in the projected coordinate system)	Spatial		–
4	Distance to the Baltic Sea coast	Spatial		–
5–16	Monthly mean air temperatures (Jan–Dec)	Meteo	E-OBS	ca. 27 km
17	Monthly mean air temperatures of previous’ year December	Meteo	E-OBS	ca. 27 km
18–21	Seasonal mean air temperatures of previous’ year	Meteo	E-OBS	ca. 27 km
22–23	Mean air temperatures of winter and spring seasons	Meteo	E-OBS	ca. 27 km
24–35	Total monthly precipitations (Jan–Dec)	Meteo	E-OBS	ca. 27 km
36	Total monthly precipitations of previous’ year December	Meteo	E-OBS	ca. 27 km
37–45	Cumulative growing degree days (GDD)	Meteo	E-OBS	ca. 27 km
	from 0 to 8 ^∘^C with an interval of 1 ^∘^C			
46	Cumulative growing precipitation days (GPD)	Meteo	E-OBS	ca. 27 km
47	Presence of snow cover (0–1)	MODIS	IMS	4 km
48–49	Consecutive number of days with and without snow cover	MODIS	IMS	4 km
50	Number of days with snow cover in a month	MODIS	IMS	4 km
51	Day of year with the last snow cover	MODIS	IMS	4 km
	NDVI, EVI, LAI, and fPAR means aggregated in AtPol 10 × 10-km grid:	MODIS	MYD13Q1 /	0.25–1 km
			MOD13Q1	
52–55	- For all available values	MODIS	– || –	0.25–1 km
56–59	- Based only on the highest pixel reliability (i.e., flagged as “0”)	MODIS	– || –	0.25–1 km
60–63	- Based only on the highest and average pixel reliability (“0–1”)	MODIS	– || –	0.25–1 km
64–67	- Based on all available except the lowest pixel reliability (“0–2”)	MODIS	– || –	0.25–1 km
68–79	- 1-week rolling mean group by pixel reliability (“0,” “0–1,” “0–2”)	MODIS	– || –	0.25–1 km
	- The rate of change grouped by pixel reliability (“0,” “0–1,” “0–2”) between:			
80–91	– Monthly and 10-day average	MODIS	– || –	0.25–1 km
92–103	– 10-day average and one-week rolling mean	MODIS	– || –	0.25–1 km
104–115	- Normalized for particular pixel’s location and	MODIS	– || –	0.25–1 km
	grouped by pixel reliability (“0,” “0–1,” “0–2”)			

#### Meteorological derived indices

The timing of plant developmental events is highly dependent on temperature, precipitation, and photoperiod conditions, and therefore, it is the most common strategy for correlating the plant phenophase with the weather conditions (Yan and Hunt 1999). To detect plant reactions to changes in the atmospheric environment, archive station measurements are normally used. However, in this study, the authors decided to use the high-resolution (ca. 27 km) E-OBS gridded dataset provided by the European Climate Assessment & Dataset (ECA&D, Haylock et al. (2008)). The application of gridded dataset instead of in situ measurements allowed to reduce any potential problems with data inhomogeneity or situations where phenological observations were done in quite a distance from the nearest measurement stations. Moreover, E-OBS dataset (Hofstra et al. 2009) assures high quality for the applied data and renders further developed phenological model assumptions usable in other European regions.

A wide group of agrometeorological indices derived from the E-OBS temperature and precipitation gridded data were used as potential predictors. In this study, we decided to calculate a set of cumulative growing degree days (GDD) from 0 to 8 ^∘^C with an interval of 1 ^∘^C (calculated from January 1st) to account for a wide range of thermal sensibilities in particular plant species. Similarly, we also took into account the different water needs of plants for different phenophases which should be reflected in the cumulative growing precipitation days (GPD) calculated from January 1st onwards. Complementary thermal and pluvial conditions were represented by seasonal and monthly air temperature averages, seasonal and monthly sums of precipitation for each month of the current and previous year. Altogether, 42 meteorologically based features were created.

#### Moderate-Resolution Imaging Spectroradiometer-derived products

Observing vegetation from space poses a number of challenges related to many sophisticated effects such as atmospheric and soil effects, pixel aggregating techniques, and observation geometry (Testa et al. 2014). All of them affect the obtained data in a different way and become especially problematic in high and mid-latitudes (Hird and McDermid 2009). Despite such limitations, many previous studies have proven that remotely sensed observations may still be a robust tool for monitoring seasonal cycle of vegetation, even in areas not particularly approachable for satellite imagery (Karlsen et al. 2008). Moderate-Resolution Imaging Spectroradiometer (MODIS) level-3 vegetation products were used for detecting onset dates of particular phenophases. The following indices were used: Normalized Difference Vegetation Index (NDVI), Enhanced Vegetation Index (EVI), Leaf Area Index (LAI), and Fraction of Photosynthetically Active Radiation (fPAR) (Knyazikhin et al. 1999; Huete et al. 2002). NDVI and EVI contain information about live green vegetation and are delivered as MYD13Q1 and MOD13Q1 MODIS products with sinusoidal projection at 250-m resolution and 16-day intervals. Using interleaved Terra and Aqua sensors simultaneously makes it possible to couple them into an 8-day temporal resolution product. Due to the rather noisy NDVI and EVI data, especially in the colder part of the year (Hird and McDermid 2009), the authors then decided to take into account pixel values aggregated within a commonly applied in a national-scale geobotanical research a 10 × 10-km AtPol grids (Fig. 1, (Zajac 1978; Komsta 2016)). To smoothen the raw MODIS data into daily time-series, a spline algorithm was applied.

The next vegetation indices, LAI and fPAR, are 1-km products provided on a daily basis (Knyazikhin et al. 1999) and were also re-calculated for a wider extent of 10 × 10-km grids. LAI was used as an index to define an important structural property of a plant canopy, namely the one-sided leaf area per ground area unit. The fPAR index measures the proportion of available radiation in the photosynthetically active wavelengths (400 to 700 nm) that a canopy absorbs (Knyazikhin et al. 1999).

Additionally, the Interactive Multisensor Snow and Ice Mapping System (IMS) 4-km daily products derived from the National Snow and Ice Data Center were chosen to detect occurrence of snow cover (Brubaker et al. 2005). In the case of detecting occurrence of snow cover, the original vegetation indices in a corresponding time-series were replaced with zeros. In situations where the surface was not visible for the MODIS sensors (mostly due to cloud cover), the original MODIS values, often providing the mean climatology, were replaced by linearly interpolated valid values from the previous and following periods.

Besides the most probable NDVI, EVI, LAI, and fPAR values for each day, the authors also distinguished a set of derivative predictors consisting of the following: normalized values of MODIS indices for every single station, raw and corrected indices accounting for different pixel reliability, rate of change in an index value between monthly and 10-day measurements, and 1-week rolling mean. A conjunction of all selected variables gives a total of 64 MODIS-derived plant phenology indices.

This set of phenological products were supported by the operational IMS snow products. On the basis of the nearest grid value to the stations’ location, five measures were calculated: occurrence of snow cover (as 0–1 binary form), consecutive number of days with and without snow cover, number of days with snow cover in a month, and day of the year with the last snow cover.

#### Spatial features

To find spatial dependencies for the analyzed locations, four geographical variables were used including longitude and latitude calculated in the projected coordinate system, altitude based on the corrected Shuttle Radar Topography Mission (SRTM-3) dataset (Reuter et al. 2007), and the distance in kilometers to the Baltic Sea coast line for each of the monitoring sites. The latter feature was added to capture local processes observed in the Baltic Coastal zone that make this area climatologically unique (Czernecki and Mietus 2017), but are not fully reflected by temperature- or precipitation-related indices. Adding this variable aimed to improve overall quality of the created models for stations located up to about 100 km from the coast line.

### Model development

Six commonly used statistical methods were tested and evaluated against the observed onset dates of the selected phenophases:

1. multiple linear regression (lm)
2. multiple linear regression with stepwise selection (lmAIC)
3. least absolute shrinkage and selection operator (lasso)
4. principal component regression (pcr)
5. generalized boosted models (gbm)
6. random forest (rf)

This study splits the previously described total number of 102 potential predictors into four sub-groups that might be applied according to the needs of statistical modeling:

(i) consisting only of meteorologically derived variables and locations’ features (meteo)
(ii) MODIS-derived predictors (modis)
(iii) all available variables preprocessed with the use of Boruta algorithm to find all relevant features (Kursa and Rudnicki 2010). The role of the Boruta algorithm is to remove features that show to be less important than a random variable (boruta)
(iv) all available variables without any preselection (all)

To avoid situations where a “future” dataset would be applied according to the needs of predictive model building, only predictors that could be calculated by the typical onset date of a particular phenophase were used. For example, *Corylus avellana* flowering phase, observed typically in March, could have been modeled with the use only of indices obtainable before and during this month. Such a solution assures that created models may also be applied as supplementary information supporting the national phenological network or for further investigation related to the spatial prediction of phenological phases.

A k-fold cross-validation strategy was used to avoid overfitting and to estimate the accuracy of the models. For that purpose, the dataset was divided into eight 1-yearly subsets (2007–2013). Next, the model was trained on seven (k-1) years, and the held-out subset (1 year) was used to evaluate the model. This procedure was repeated eight times. The overall performance was obtained by averaging the k estimates of the performance (Kuhn and Johnson 2013).

The models’ performances were characterized using the coefficient of determination (*R*^2^) and root-mean-square error (RMSE). An *R*^2^ value is the squared correlation coefficient between the observed and predicted values. RMSE is the difference between predicted values and observed values. Additionally, the model’s distribution errors for selected cases were presented as histograms and scatterplots.

The general effect of the independent variables on gradient boosted models was determined using a variable’s “relative influence” (Friedman 2001). Values of variable influence/importance were obtained separately for the models based on all data from each phenophase. The ten best predictors were then selected and divided into meteorological, MODIS-derived, and spatial categories. Afterwards, for each category of predictors, the mean variable importance was calculated and scaled so as to estimate which predictors contribute in the highest degree to a model’s prediction (Fig. 6). All calculations were carried out using R programming language (R Core Team 2016) and its packages such as “Boruta,” “ranger,” or “caret” supporting machine learning techniques (Venables and Ripley 2002; Kuhn 2008; Kursa and Rudnicki 2010; Wright 2015).

## Results

Ground observations of 10 plant species yielding 13 phenophases (Table 1) at 52 phenological stations in Poland were analyzed. Their temporal range varied from the earliest spring (flowering of hazel occurring on 70 days of the year on average), to late autumn (leaf falling of silver birch occurring on 301 days of the year on average) (Fig. 2). Additionally, the timing of phenological phases significantly differed between species, both in space and in particular years. The largest standard deviation was found for the flowering of hazel (about 17 days) and the smallest one was observed for the horse chestnut’s flowering (about 6 days) (Fig. 2). In general, the characteristics of phenological phases presented in this study stay in agreement with very detailed (i.e., based on larger number of stations) nationwide phenological patterns observed in the past decades (Tomaszewska and Rutkowski 1999), although the acceleration of particular phenological phases is clearly visible and follows trends described in other research studies for this part of Europe (Menzel et al. 2006; Czernecki and Jabłońska 2016; Templ et al. 2017).

### Models’ performance

**Fig. 3:**
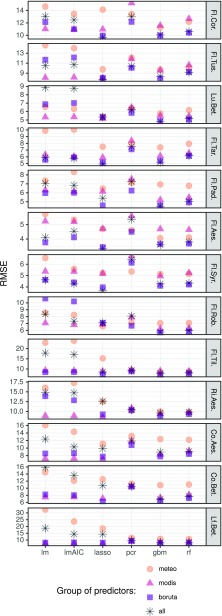
Performance of models of 13 phenophases using four groups of predictors. Phenological stages sorted in increasing order (i.e., with the earliest phases on the top)

Calculation of 416 final models (13 phenophases × 6 modeling techniques × 4 groups of predictors) was carried out (Fig. 3). Final models varied distinctly between modeled phenophases, as well as between tested modeling techniques and group of predictors used. On average, horse chestnut flowering and lilac flowering models gave the best RMSE values of about 5 days. In contrast, the largest RMSE values of about 12 days were obtained for silver birch leaf falling and flowering of hazel. Standard deviation of RMSE was between about 1 (horsenut flowering) and 3 days (silver birch leaf coloring) for most of the models. Distinctly higher numbers were noted for the flowering of small-leaved lime (5.5 days) and birch leaf falling (7 days).

Models were built using four groups of predictors: (i) meteorological, (ii) MODIS-derived, (iii) filtered predictors using the Boruta algorithm, and (iv) all groups combined. On average, RMSE values were 7.6 for MODIS-derived predictors, 7.7 for predictors preselected using Boruta, 9.1 for all predictors, and 11.0 for meteorological predictors. Their standard deviations were respectively 2.1, 2.6, 3.9, and 5.6. The impact of the predictors’ groups was different for each taxon and model. MODIS-derived predictors proved to be the best in models of horse chestnut fruit ripening, horse chestnut leaf coloring, and leaf falling of silver birch. The best models of black locust flowering, small-leaved lime flowering, and silver birch leaf coloring were based on predictors preselected using the Boruta algorithm. In the rest of the phenophases (flowering of hazel, coltsfoot, dandelion, hackberry, lilac, and leaf unfolding of silver birch), all predictors combined were used. Only the meteorologically derived predictors were not used in the best model.

On average, multiple linear regression models gave the worst RMSE value of around 10 days (Fig. 3). Multiple linear regression with stepwise selection and principal component regression average value was only slightly lower at 9.5 and 9, respectively. However, the worst average models differ between phenophases. Multiple linear regression models were the worst for seven phenophases, multiple linear regression with stepwise selection for four phenophases, and principal component regression for two phenophases. Generalized boosted models, random forest, and lasso have the lowest average values of RMSE, between 7.25 and 7.89. In four of the phenophases, generalized boosted models were the best, and lasso had the lowest average RMSE in the models with nine phenophases. Additionally, the impact of predictors’ group varied between modeling techniques. The choice of predictors had the smallest impact in the case of pcr, random forest, and gbm; a medium impact on the lasso model; and a large impact when applying the linear regression techniques (i.e., lm and lmAIC models) (Fig. 3).

### Best models

**Fig. 4:**
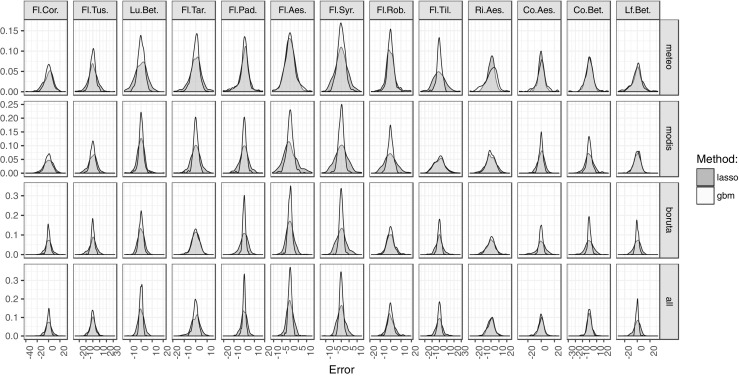
Distribution of errors in generalized boosted models and lasso models of 13 phenophases using four groups of predictors

Generalized boosted models and lasso were proven to have the best prediction of the studied phenophases (Fig. 3). Distribution of errors in these models was compared for all phenophases and predictors groups (Fig. 4). A direct comparison showed that errors of lasso were either very similar to those of the generalized boosted models or more widespread around zero (perfect) value. Conversely, the distribution of errors in gbm had smaller tails and was more dense around zero. The biggest differences between models were for the silver birch leaf unfolding, and the flowering of hackberry, horse chestnut, and lilac.

**Fig. 5:**
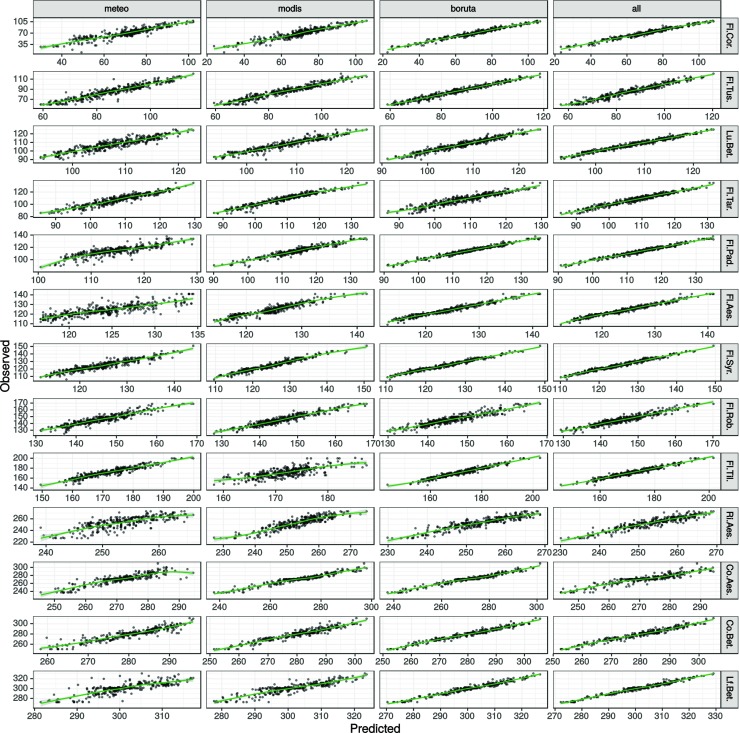
Relationship between observed and predicted dates for generalized boosted models of 13 phenophases using four groups of predictors. Values given as days of year

Relations between predicted and observed values of gbm showed a visible distinction of the used predictors groups (Fig. 5). Overall, predictions of gbm based on meteorological variables or MODIS-derived variables were less accurate than those preselected using Boruta or based on all data. The quality of meteorologically based gradient boosted models was comparable only in the case of flowering of dandelion and black locust. MODIS-derived predictors worked especially well for gbm in the case of horse chestnut leaf coloring. Predictors filtered using the Boruta algorithm gave the most stable gradient boosted models for flowering of coltsfoot and leaf coloring of horse chestnut, while all data worked best for the leaf unfolding of silver birch and dandelion flowering. In the rest of the phenophases, prediction based on all data or preselected by the Boruta algorithm proved to have similar accuracy.

### Variable importance

**Fig. 6:**
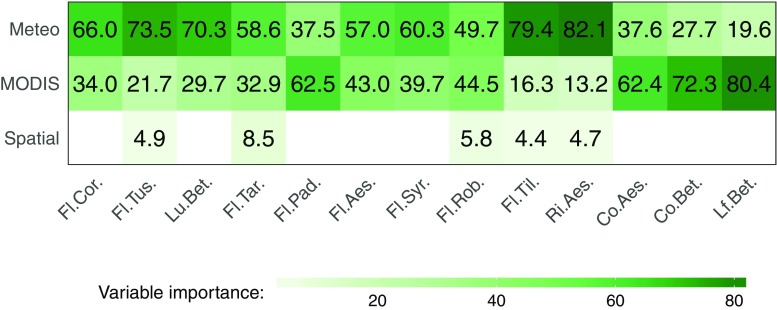
Variable importance according to group of predictors applied in generalized boosted models (gbm). For each phenological seasons values scaled up to 1 (values given in %). Detailed description in the “Model development” and “Variable importance,” and Table 2

Gradient boosted models based on all data gave better accuracy than models created using only meteorological or MODIS-derived predictors. Therefore, calculation of any variable importance was performed for the models based on all data. A variable’s “relative influence” (variable importance) was obtained for the top ten predictors of gradient boosted models for each taxon. Afterwards, predictors were separated into three groups (meteorological, MODIS-derived, spatial), averaged, and then scaled (Fig. 6). The spatial group of predictors had very small impact on the models probably due to being closely correlated with meteorological variables. Only in 5 of the 13 models were the location variables included in the top ten most important variables, and their influence in most cases was limited (i.e., between 4.4 and 8.5%).

On average, MODIS-derived predictors were the most important for autumn and late autumn modeling cases (leaf coloring of horse chestnut and silver birch, and leaf falling of silver birch) with one exception: flowering of hackberry which is usually observed in April. In all other phenological phases, meteorological variables proved to be the most influential factors for phenological modeling needs.

In general, MODIS-related metrics provide more information for phenological modeling compared to the traditional meteorological indices (see Figs. 4 and 5). For example, the most promising gradient boosted models based only on meteorological data have RMSE of 8.4 days, while for MODIS vegetation metrics, this value decreases to 7.4 days. If all available datasets are together overlapped and preprocessed with the Boruta algorithm, the RMSE value decreases to 6.4 days.

## Discussion

The subjective nature of ground-based phenological observations has always been an issue in contemporary phenological research (Schaber and Badeck 2002; Fisher et al. 2007; Scheifinger and Templ 2016; Templ et al. 2017). Numerous attempts to cover the gaps by means of airborne sensors and empirical-statistical models that take into account plant sensitivity to temperature, precipitation, and photoperiod indices showed that this problem has yet to be entirely solved (Studer et al. 2007; Fisher and Mustard 2007; Fisher et al. 2007; Almeida et al. 2012). However, identification of the optimal set of factors and selecting the most robust modeling techniques can offer useful approximation of the day of phenological phases’ occurrence. In this study, satellite and meteorological products were used as predictor variables to build models for the reconstruction and prediction of the selected phenological phases in Poland. Due to relatively limited spatio-temporal coverage of the available dataset (4524 observations), the cross-validation part was taken with a special care to avoid overfitting and to ensure model applicability also for past and future conditions. A relatively small differences between cross-validation steps confirms that the developed methodology of phenological modeling based on simultaneous application of meteorological and satellite products may be universal for all areas sharing the same plant phenological phases and show a high potential in combining the extensive knowledge generated by the ground-based phenological modeling community to satellite data (Fisher and Mustard 2007).

The machine learning algorithms used in this study (Kuhn 2008; R Core Team 2016) are available free of charge. They can be run on any modern desktop computer usually in a time below a few seconds, depending on a selected model and algorithm’s parallel capabilities. Data preprocessing and feature engineering of input data (i.e., downloading, cropping, reprojecting, cleaning, reshaping, calculating indices, etc.) are the most time-consuming tasks in getting ready-to-use models; however, they are crucial for creating the final database that might be easily applied for a model’s evaluation. The cross-validation procedures are also quite computationally demanding. In this case, several hundreds of a single-core computational hours were required in order to avoid overfitting, and therefore, application of high-performance computing clusters was needed. However, these procedures need to be run once before the deployment of models in an operational mode.

The created machine learning models show varying accuracy of reconstructing and predicting particular plant phenophases. The model biases given by RMSE values are in some cases related to the range of possible onset dates of particular phenophases (see Figs. 2 and 3). This is clearly seen in the case of earliest spring and autumn phenophases whose standard deviations of onset dates and RMSE values obtained for the best models are usually relatively high (7–10 days) compared to other seasons.

Due to the nonlinear reaction of plant species to thermal (Sparks et al. 2000; Iler et al. 2013; Jochner et al. 2016) and photoperiod conditions (Cober et al. 2014), the simplest models based on regression techniques were in most cases not as robust as more sophisticated gradient boosted, random forest, and lasso models. However, while applying a correctly specified (preprocessed) set of predictors, the differences between particular family of models became smaller with the average total RMSE for all phenophases in the range of 8.3 (multiple linear regression) to 6.4 days (generalized boosted model). The commonly applied preprocessing approach, such as the AIC stepwise screening (Sakamoto 1992), hardly influences the obtained results and, in authors’ opinion, does not redress the computational time required for applying this procedure. Significantly better preprocessing results were obtained by the Boruta algorithm (Kursa and Rudnicki 2010) which also reduced the computational time needed for running the created models.

Despite numerous deficiencies in such an approach and clear limitations of applying modern satellite observation in plant phenology modeling, it might still be able to give a reliable proxy for traditional ground observations, especially in terms of early spring and late spring phenophases for the best models whose calculated RMSE is below 3–4 days. However, the calculated contribution of each variable showed rather small or at best moderate influence of satellite-derived products (especially in terms of early phenological stages), even though higher for meteorological features. Better fit of meteorological features in case of spring phenological phases proves significant temperature dependence for early spring season in opposite to autumn season (Jabłońska et al. 2015). The influence of temperature on plant growth is definitely higher in the spring, when they start their development cycle after the winter break than in autumn, when already mature plants are less sensitive to temperature fluctuations. The largest RMSE values for flowering of hazel and silver birch leaf falling correspond to the seasonal dynamics of those phenological phases in Poland. Each phenological season is characterized by specific variability. Higher standard deviation for early spring and autumn seasons is a characteristic feature observed throughout Europe, which is connected with more variable solar radiation receipts then (Schwartz 2013).

This regularity is also reflected in remote sensing data, which tend to contain noisy information (Hird and McDermid 2009), and thus are often omitted when applying preprocessing procedures for modeling of spring and summer phenophases. Therefore, most of the created phenology models are primarily based on meteorological metrics (i.e., usually GDD) with only slight improvements when using satellite-derived products. This situation changes in favor of airborne sensors for late autumn phenophases where the calculated variable importance of this features varies from 62.4 to 80.4% (Fig. 6). On average, the application of remote sensing products improves the accuracy of created phenological models for 1.8 days in terms of obtained RMSE values, and in the case of autumn phenophases for 2.9 days.

The improvement in the models’ performance for later phenophases when applying satellite vegetation products should not come as surprise as the physiological plant reaction to atmospheric conditions is related more to summer and autumn seasons’ conditions than for instance GDD that are usually calculated from the beginning of the year, and thus, may not reflect the most recent autocorrelation signal, lagged by 1–2 months. The analysis of national phenological trends in Europe proved that air temperature in the autumn does not have such a clear effect on coloring and falling of leaves (Menzel et al. 2006). It must also be remembered that the applied aerial approach with aggregating sparse areas near to the station’s location covers usually a complex mosaic of different plant species with significant contribution of green and forest areas. These ecosystems clearly react in the autumn season via changing leaf pigments and are thus sharply reflected in changes in NDVI and EVI values, providing a clear and valuable signal for phenological models.

## Conclusions and future work

Historically, most of statistical and mechanistic phenology models *were developed for tree species, rather than non-woody species* (Chuine et al. 2013). Despite huge progress in phenological modeling in the recent decades, the potential of modeling non-woody and non-agricultural plants with estimation of cross-scalar phenology was still underestimated or applied sporadically (Fisher and Mustard 2007; Xin et al. 2015). The approach presented in this paper shows the moderate-to-high potential of using machine learning models to fill temporal and spatial gaps in ground-based observations as well as forecasting selected phenological phases by means of remotely sensed and meteorologically based products. This allows for the possibility of reconstructing the Polish phenological dataset for the years 2000–2005 using the MODIS vegetation products, while Landsat scenes may be used for the missing period of 1994–1999, as they give comparable results to the MODIS products (Fisher and Mustard 2007).

In the authors’ opinion, the developed strategy shows potential for estimating phenology from remote sensing using machine learning algorithms. It could be done by applying larger database and thus spatio-temporal extension of cross-validation periods that would include a wider response to climatological forcing. There is also a possible improvement in application of other satellite-derived products, especially if they are complementary to rather low time frequency of MODIS vegetation products. To some extent, the created models also indicate factors that are responsible for accurate modeling of particular phenophases and help in better understanding of vegetation dynamics to climate variability, even though it must be kept in mind that non-mechanistic approach may fail in terms of facing a physiological limits of particular plants (Zhao et al. 2013). This limitations certainly need to be addressed if cross-scalar machine learning (i.e., statistical) models would be used in improving national networks of phenological observations.

The created empirical-statistical phenological models may not only be used as a reliable proxy for ground-based measurements but may also have the applicable potential for operational forecasting needs. Moreover, since aerobiological data have a high correlation with plant phenological phases (Kasprzyk 2003; Estrella et al. 2006; Bogawski et al. 2014), further improvements in airborne pollen allergy modeling may benefit from the synergy of satellite and meteorolo gical data, as well as machine learning algorithms.
